# Does the Presence of Asbestos-Containing Materials in Buildings Post-construction and Before Demolition Have an Impact on the Exposure to Occupants in Non-occupational Settings?

**DOI:** 10.7759/cureus.37305

**Published:** 2023-04-08

**Authors:** Raja Singh, Arthur L Frank

**Affiliations:** 1 Architecture, School of Planning and Architecture, New Delhi, IND; 2 Built Environment and Public Health Research Fellowship Program, Tathatara Foundation, Bobbili, IND; 3 Centre for Built Environment Policy, Information Sharing and Analysis Center (ISAC), New Delhi, IND; 4 Environmental and Occupational Health, Drexel University College of Medicine, Philadelphia, USA

**Keywords:** exposure guidelines, healthy buildings, pleural mesothelioma, mesothelioma, asbestosis, indoor air quality, airborne asbestos, non-occupational exposure, building materials, asbestos

## Abstract

This narrative review aims to determine if asbestos-containing materials in buildings pose a hazard to building occupants in non-occupational settings. This paper is limited to the post-construction and pre-demolition stages of a building. The researchers selected 19 studies from the 126 studies screened, concerning exposure to asbestos fibers in non-occupational building settings, with a focus on post-construction and pre-demolition phases. The literature review found that certain conditions, such as the measurement techniques, standards, and previous data availability, prevent a conclusive answer to the research question. Some studies have pointed towards an effect of asbestos-containing materials on health of occupants in non-occupational settings. But, there are some that do not suggest a positive relationship between non-occupational exposure and the presence of asbestos-containing materials, and therefore these provide scope for further research, as these studies also do not rule out the relationship completely. The present study highlights the gaps in current knowledge and indicates areas for further research. Until conclusive evidence based on revised threshold standards and accurate measurement techniques is available, asbestos-containing materials may be considered unsafe for use in non-occupational settings, especially ones that young people and children occupy.

## Introduction and background

Asbestos is a mined mineral composed of long, thin fibres that are heat-resistant and have been used by humans in a variety of commercial and industrial applications, such as insulation, fire-proofing, and cement composite products. It has found extensive use in the building and construction industry. This cheap and useful material may have good fire resistance properties and may act as a good insulator, but it is, in fact, a toxic material for humans. Millions of lives across the world have been taken due to asbestos and its effect on human health. In mining, processing, manufacturing, installation, demolition, and disposal, asbestos fibers are released into the air. In the absence of protection, which is more often than not, rarely provided, the asbestos fibers enter the lungs and cause either non-malignant diseases like asbestosis, pleural effusions, etc., or malignant conditions like mesothelioma, lung cancer, and other forms of cancer [1]. A damaged lung can also lead to further complications.

The World Health Organization (WHO) has urged stopping the use of all types of asbestos, as none is safe, whether it is amphibole or serpentine fibers. Many countries have banned its use, but others have “exported” its manufacture and use to developing countries. Some countries in the world actively mine, process, and use asbestos for manufacturing, while others have banned mining, but the use of imported fibers for processing and manufacturing is still prevalent. One such example is India, where like many other Asian countries, 90% of asbestos is used in asbestos cement-based sheets and pipes. However, the mining ban has been challenged.

Legislative or executive action to stop the manufacture may create a parallel industry or put its use in the blind spot of enforcement agencies. This means that an illicit manufacturing industry in the country or the smuggling of asbestos products from jurisdictions with no prohibition may take place. Therefore, while dealing with asbestos, an approach to stopping its manufacture may be by banning its production along with discouraging or inhibiting its consumption for application as a final processed product. This can happen through policy-level intervention or public awareness so that the end customer demand for the product reduces in the market leading to the automatic erosion of its supply, and and eventual stop in its use. If its use as the final product can be stopped, its production will stop without any legal or executive action. Policy-level intervention means removing asbestos from building bylaws, building specifications, construction industry manuals, and architectural education curricula. But first, evidence needs to be strengthened, by including the most recent studies, on whether using asbestos-containing materials in non-occupational situations poses a threat to health by creating exposure to asbestos fibers in buildings. 

There has already been considerable interest in studying the effect of asbestos in situations where it poses an occupational threat, and there is evidence that occupational use is harmful to health and can be fatal. This included asbestos mining, asbestos processing, or asbestos products installation. On the other hand, the effect of non-occupational and environmental exposure to asbestos is also of interest, and studies have ventured to investigate this aspect and have found evidence of diseases. Our focus in this review is on this aspect and can be stated as follows: Does the presence of asbestos-containing materials in buildings post-construction and before demolition have an impact on the exposure to occupants in non-occupational settings?

Aim

To determine, using a review of peer-reviewed literature, whether the presence of asbestos-containing materials in buildings post-construction and before demolition impacts exposure to asbestos and the health of occupants in non-occupational settings

Methods

Eligibility Criteria

The quest was to find out what is known about non-occupational exposure to asbestos and its link to human diseases. This non-occupational risk has been of considerable attention in the 1990s due to certain incidences in New York schools and elsewhere. But as is seen, there have not been many studies when the scientists have considered this research problem (i.e., the non-occupational effect of using asbestos-containing materials in the buildings as a building material). 

Excluded from this study were occupational studies that included people who have directly worked in the mining industry or have any work or occupational exposure to asbestos. It is almost universally accepted that occupational exposure to asbestos leads to diseases affecting the lungs and often asbestosis. Our specific interest was on the non-occupational part as some countries have banned asbestos, which is limited to mining bans and or processing bans, but the use of asbestos as roofing sheets is still prevalent. Some examples are countries in Asia that consume 85% of the world’s asbestos. It must also be noted that 90% of asbestos is used in cement sheets and pipes.

Even if using materials containing asbestos is banned, it is crucial to understand that the ban will be prospective and not retrospective. There will be many buildings that will still contain asbestos. Their demolition may be a one-time activity and can even be under controlled conditions, but their active use cannot always be accompanied by people wearing precautions for everyday activities. The risk in such buildings using asbestos-containing materials had to be ascertained, and such information should be available for policymakers to make informed decisions.

Information Sources

The results of the specific questions from the Problem-Intervention-Comparison-Outcome (PICO) analysis, as given in Table 1, yielded very few results on PubMed. Hence the keywords were broadened to find the relation between asbestos and buildings. Filtering was done when there were sufficient results available for study. The search was done in January 2023 using PubMed Database, which is a reliable source of literature with verified journals. The filter used for additional search included Free Text Available. The search terms used in PubMed, along with the Medical Subject Heading (MeSH) terms, are given in Table 2 below.

**Table 1:** The Problem-Intervention-Comparison-Outcome (PICO) Analysis of the Problem

PICO components	Description
Problem	Asbestos is integrated into buildings, increasing the demand for asbestos.
Intervention	Asbestos is removed from building specifications, regulations, bylaws, and national standards in India.
Comparison	Building regulation-level intervention for universal accessibility has yielded results.
Outcome	Relation between buildings and asbestos will be available, Whether the presence of asbestos in buildings post-construction and pre-demolition have a long-term impact on occupants? This will prevent health hazards.

**Table 2:** The Search Terms for Advanced Search in PubMed MeSH: Medical Subject Headings

Serial Number	Search Terms	Expansion
1	(asbestos) AND (buildings) Filters: Free full text	(("asbestos"[MeSH Terms] OR "asbestos"[All Fields]) AND ("build"[All Fields] OR "building"[All Fields] OR "building s"[All Fields] OR "buildings"[All Fields] OR "builds"[All Fields])) AND (ffrft[Filter])
2.	Translations	asbestos: "asbestos"[MeSH Terms] OR "asbestos"[All Fields] buildings: "build"[All Fields] OR "building"[All Fields] OR "building’s"[All Fields] OR "buildings"[All Fields] OR "builds"[All Fields]

**Figure 1:**
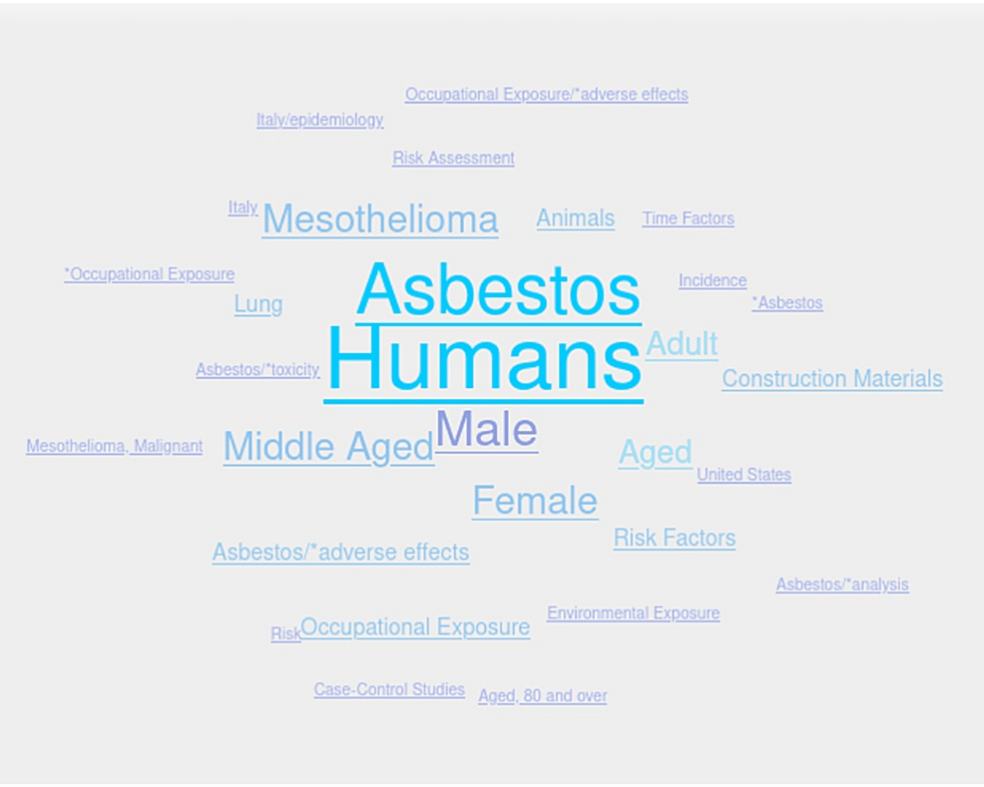
The Word Cloud of the Topics of the 126 Papers Screened From PubMed

The search yielded 126 results. The metadata of the 126 papers was exported from PubMed into Rayyan Systematic Review Software to screen the 126 papers. In Rayyan, a Word Cloud was also generated, which is given in Figure 1. 

The 126 papers were screened using Rayyan by reading the title and the abstract [2]. In the review, selection was based using inclusion and exclusion criteria to select the papers further. 

Inclusion Criteria

The study included papers on buildings, building materials, building design, non-occupational exposure to asbestos fibers in buildings, and the post-construction and pre-demolition phase of a building. 

Exclusion Criteria

The research excluded studies on occupational exposure to asbestos and papers published from 1992 to 2022. This was done to get the most recent evidence as older technology has been replaced with newer methods, protocols, and permissible limits. Papers that did not primarily deal with buildings or built environments and were concerned with mines or other environmental exposure were also excluded in the current study. 

**Figure 2:**
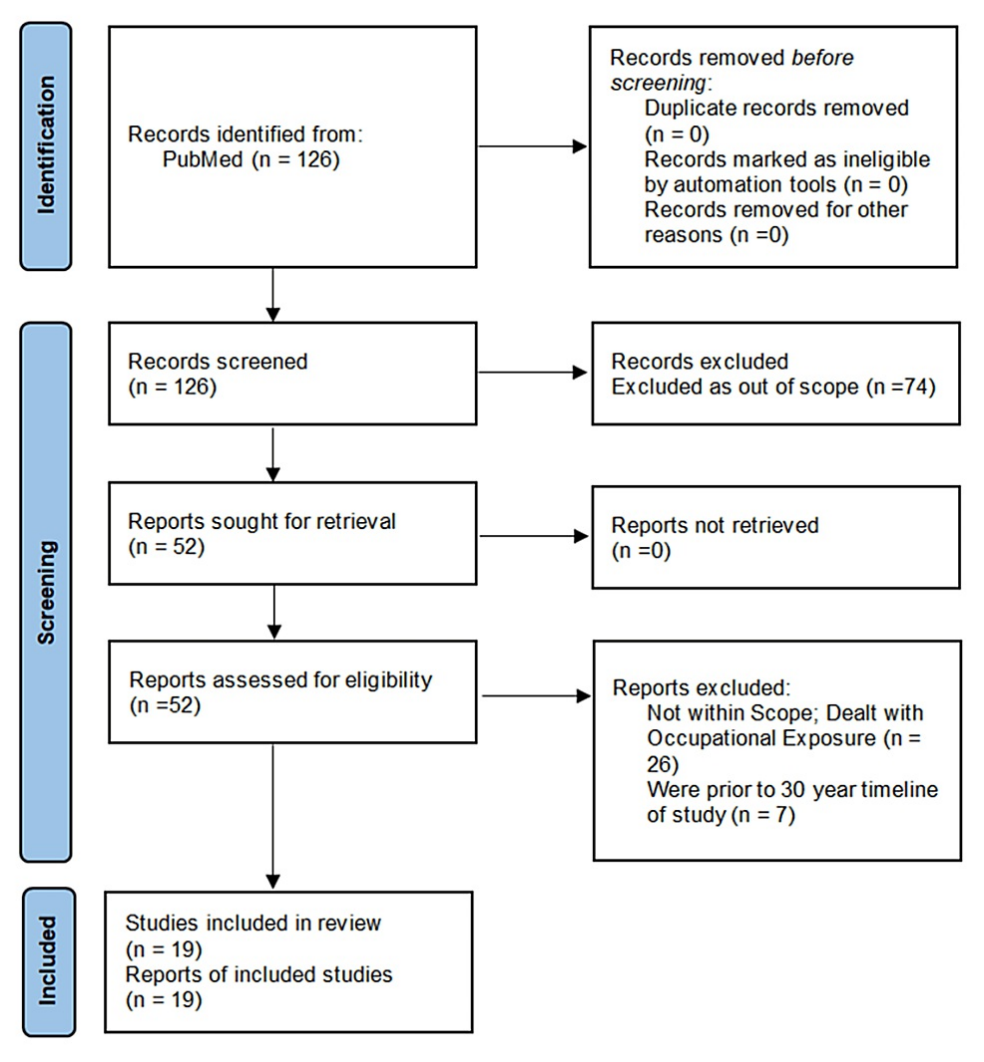
The Flow Diagram of the Records Identified, Screened, and Included Source: Author As per the format of PRISMA Statement 2020 [3] PRISMA: Preferred Reporting Items for Systematic Reviews and Meta-Analyses

The present research used papers available for free access as a filter. The flow diagram of the screening of the papers is shown in Preferred Reporting Items for Systematic Reviews and Meta-Analyses (PRISMA) format, with a statement of identification of studies via databases and registers [3]. The flow diagram is given in Figure 2 below. 

This study is exempt from any ethics review for multiple reasons. First, according to the National Ethical Guidelines for Biomedical and Health Research Involving Human Participants (2017), systematic reviews are exempted from ethics review [4]. Second, this study did not include human participants, human tissues, and animals. There was no questionnaire, and no person was approached for this study.

Limitations of the study

This narrative review is limited, given the search terms used. As a narrative review, it provides a summary or overview of the topic, with a broad focus and the sources of literature may be non-exhaustive. This, among other points, is a limitation inherent to narrative reviews. The included studies were the available ‘Free Full Texts’ on PubMed. Some studies which may have been relevant may have been excluded. Another limitation may be that the time of the selection has been limited to three decades before 2022. There may be studies before 1992 that have not been included. This was done to bring in the most relevant papers that use the updated protocols, permissible limits, and technologies of asbestos analysis.

## Review

The 19 records included were thoroughly reviewed, and their basic descriptions are detailed in Table 3 in the Appendix.

The earliest study we have included was from Israel in 1992, where a dining hall from a kibbutz was studied [5]. It was found that the asbestos levels were higher than guidelines and suggested further research in all buildings where asbestos-causing materials are used. This was followed by a study performed in 1993, which not only reinforced the fact that all forms of asbestos harmed humans but also warned of asbestos-containing materials in buildings and how it may cause continued exposure [1,6]. But they did not investigate empirically or did not provide the framework or investigation of the same at that time. 

On the opposite end, two studies shunned the need for any interventions in buildings that may contain asbestos-containing materials unless there was some repair being performed in the building [7-8]. Another British study in 2018 also stated that the environmental damage caused by asbestos is negligible but left space for further studies, especially in school environments where asbestos-containing materials may still be used [9]. 

Since there are concerns about asbestos fiber release during building repair, a study was performed with building maintenance personnel in 1996 [10]. The study started with the fact that there will be concerns for building maintenance personnel and stated that if operation and maintenance protocols are met, the asbestos exposure risks faced by the personnel will decrease. In another Japanese study, there were links to asbestos exposure and tobacco exposure, but not a particularly quantitative assessment [11]. 

An important study was performed in 2009 in Poland, where city-level environmental exposure was studied [12]. It was found that mapping can be performed in a city to locate hot spots. Additionally, the study stated that wind velocity, even at low levels, disperses asbestos fibers from the source, which may be a building with asbestos cement sheets. The study also states that there was a basic lack of data in the literature on the urban and rural levels of ambient asbestos levels, and countries like India may not have the required data.

In another study, a case of a patient was taken. A 26-year-old person was diagnosed with mesothelioma, and it was found that the only exposure was of six years at school, where there was asbestos-containing materials [13]. This hints at the link between exposure in non-occupational settings, but the study had some shortcomings. The absence of control in the study and the expansion of the sample was needed, with more people attending school with the patient needing to be tested. But, based on this, we cannot rule out the non-occupational risk of asbestos fibers in the post-construction and pre-demolition phases of a building. 

Another included source was a review article as it details this issue very extensively. It discusses multiple non-occupational asbestos exposure studies with a focus on the health effects [14]. This source brings to light some very important discussions. The first is that there is no epidemiological data directly related to possible risk, but the study concurred that it would not mean that the harm can be ruled out. The paper also suggested an alternative approach which states that since the incidence of mesothelioma in the absence of asbestos exposure cannot happen, most cases of mesothelioma, which are not exposed to occupational asbestos, may be linked to non-occupational exposure. 

In 2014, another study compared various literature sources to bring forth the fiber size and its links to toxicity [15]. As it is usually believed, the mere conclusion that fibers above five microns alone are linked to adverse health effects may not be appropriate, as asbestos fibers below five microns, called short asbestos fibers or SAF, may not only be a good but ignored indicator of asbestos-containing materials but can an adverse health effect indicator, especially if indoor air quality is concerned. The study discusses that the measurement of the past may need replacement by a newer method and reassessment of the five or more-micron standard, which is above the length for asbestos fibers. 

A review paper written in 2016 stated that large corporations indirectly had given way to outsourcing asbestos industries from developed to developing countries [1]. The study warned of the need in Asia, which is currently consuming 85% of the world’s asbestos, to mitigate asbestos-caused diseases. It also stated that 90% of asbestos was used in cement sheets or pipes. The study also warned about the lobbying by the asbestos industry and the fraudulent practice of introducing doubt science, which creates a narrative that certain asbestos may be safe or creates some divergence from the WHO-stated mandate, which insists that all types of asbestos should not be used. 

Another interesting study from Australia reviewed the history of asbestos-related issues and stated that the first wave was due to asbestos mining [16]. The second one was due to using asbestos products, and it was speculated that the third one might be due to ambient exposure using products and building with asbestos-containing materials. The study also stated that cases were occurring in Australia and advocated for removing asbestos from buildings as a sensible idea. 

A more recent Swiss study performed in 2019 highlighted some important points [17]. It stated that there is a need to prevent and monitor asbestos use in schools and environments that young people inhabit. It also emphasized a procedural improvement by indicating that averages may not be appropriate as there may be general ambient readings and other event-based readings that must be taken separately. The general exposure readings may differ from event-based readings, such as when a board containing asbestos materials fell from the ceiling, etc. The study also challenged the threshold of 1000 fiber/m3 and stated that amosite-based fibers might need lower thresholds. The limitation of the study was that the dose-response was based on occupational exposure to adults, and these extrapolations may not be accurate, especially for young students in a school. The study pointed out a gap-the need to have more quantitative health-risk assessments that can be used as evidence to guide policy. This was further strengthened by a Belgium review study in 2020, which stated that the basis for the values of asbestos could not be ascertained as there was not enough monitoring data available [18].

A study in Korea attempted to specifically work on the risk caused by asbestos roofing and used past studies collation and calculate risks with corrugated asbestos roofs used in buildings [19]. The study stated that the risks of cancer throughout the lifetime were moderate or low on indoor exposure and even during the slate dismantling. But the study nevertheless stated that a gap still exists in finding carcinogenicity, and the actual exposure studies need to be performed by actual measurement if exposure to non-occupational asbestos fiber exists. The effect of such roofing on the soil was also seen in another study, where the asbestos fibers get deposited through the drainage in the roofing and eventually gather around the gutter drain, where it may not fully degrade over time and may be of risk to humans if soils are eroded or not treated [20]. Another study in Korea brought in evidence to suggest the non-occupational risk of asbestos fibers. It was evident in the cohort studies there were asbestos-related diseases caused to workers who were not linked to any occupational exposure but were in the accommodation and food industries where the exposure can only be non-occupational, and it may be due to the use of asbestos-containing materials in the buildings or the products/technology used by workers [21]. 

The latest study brought in some conclusions. It stated that the mere level of dust pollution in buildings with asbestos materials that are actively used is above normal [22]. It recommended that children and young people should not use such buildings. It went on further to state that generally, ventilation helps reduce asbestos dust over time. On the question of whether the asbestos in buildings containing asbestos materials should be removed, the study suggested that if there is no evidence of increased airborne asbestos, the removal from the building materials can be postponed. The study also brought forth the distinction in the types of constructions using asbestos materials. It stated that buildings that are non-rigid, like the ones with steel frames and asbestos as sheds have higher airborne asbestos levels than rigid ones using concrete, which may suggest that agitation leads to more disturbance and hence more release of asbestos fibers. 

The way forward is summed in five points. Firstly, further studies may focus on the environmental exposure data for countries where this data may not have been collected [14]. Secondly, replicating studies with more accurate measurement techniques, such as Transmission Electron Microscopy or TEM instead of Phase Contrast microscopy, and creating standards for the use of TEM may be further performed. The length of five microns may also be challenged as it may have an arbitrary existence in the first place [15]. Thirdly, corrugated sheet roofing has already been gaining attention [23-25]. Studies on asbestos sheet roofing, prevalent in Asia, may be performed to further the understanding on the effect by measurement in cases of non-occupational risk. Fourthly, studies replicating the effect on ventilation in buildings with asbestos-causing materials may be further performed [19]. Lastly, studies providing dose-response models in general environments and not derived or based on occupational risks with increased measurement data may be necessary to provide data for non-occupational settings [8]. 

The non-occupational stock of knowledge concerning asbestos exposure and its link to disease has gaps, and these must be filled to save the large number of people who may be at risk of exposure and who may be unaware of the same. 

## Conclusions

This study set out to find whether asbestos-containing materials in buildings post-construction and before demolition impact occupants in non-occupational settings.

Most sources included in this review suggest some relation between using asbestos-containing materials in buildings in non-occupational settings and its health effect. The studies showing such lack of relation also suggest scope of further research or enhanced methods. The most recent study showed evidence that certain construction types may release asbestos fibers if asbestos-containing materials are present. Noteworthy is that sources are pointing out a relation between the presence of asbestos-causing materials and link to exposure and health effect and none of the studies have clearly absolved the possibility of risk. The studies have suggested a possibility of a gap in measurement technique or the current threshold levels or a more thorough measurement over a longer period. There are concerns about very scarce data available on lifelong cumulative exposure of individuals in non-occupational settings. It cannot be simplified to say that this is negligible enough for not causing health effects including cancer.

## Appendices

**Table 3:** List of the 19 Selected Studies, from 2022 to 1992, with Descriptions

S. No.	Details of the Study	Study Year and Location	Description of the Study Results	Remarks
1	Obmiński A [20]	2022	There were indications of asbestos that would, from the roofing, get deposited through the gutter into the soil, thereby creating soil pollution, which may not fully degrade over time and may be of risk to humans if the soil is left eroded by air and not treated. Deposits could also be carried to the insides of houses and be a risk.	
2	Obmiński A [22]	2022; East European (Polish)	The study shows that active behavior in buildings with asbestos is a cause of above-normal dust pollution. Children and young people should not use such buildings. Ventilation helps in reducing asbestos dust over time. If there is no evidence of increased airborne asbestos, the removal of the asbestos material can be postponed. Buildings that are non-rigid (steel or non-concrete) have higher airborne asbestos.	
3	Kim EA [21]	2021	This study brought to light the presence of asbestos-related illnesses not only among workers exposed occupationally (direct or indirect) but also among workers in the accommodation and food industries.	This means that the exposure may be due to the use of asbestos materials in the building or the products/technology used by workers who may conventionally not be linked to occupational asbestos exposure.
4	Lee ES, Kim YK [19]	2021; Korea	This study used past studies to collate and calculate the risks associated with asbestos roofs used in buildings. The risk of cancer throughout a lifetime was low to moderate on indoor exposure, even during slate dismantling. However, carcinogenicity and actual exposure need to be evaluated further by actual measurement studies because of the gap in research.	The study’s content can be considered for further research in two avenues—finding out the actual exposure over a longer term with cumulative measured effect of asbestos cement roofs and focusing on risk evaluation for carcinogenicity.
5	Brouwere KD, Bautmans B, Benoy S, et al. [18]	2020: Belgium	This paper specifies the indoor air quality standards for Belgium. It also states that the basis for the values of asbestos could not be ascertained as there was not enough monitoring data available.	
6	Vernez D, Duperrex O, Herrera H, et al. [17]	2019; Switzerland	The study brings forward many points, including the need for prevention and monitoring of asbestos in school and young people’s environments. There is a way of taking readings as background and event-based. General exposure was different from exposure that happened when a board fell or when building maintenance took place. The threshold of 1000 F/m3 for the general population is arguable, and amosite-based fibers may need lower thresholds.	The study had a limitation since the dose response was based on occupational exposure to adults, and the extrapolations may not be accurate. Further, using evidence-based policy can be achieved through quantitative health risk assessments.
7	Gilham C, Rake C, Hodgson J, et al. [9]	2018	The study states that the environmental damage caused by asbestos is negligible, but further studies are warranted to check the same in schools.	
8	Armstrong B, Driscoll T [16]	2016	The study speculates on the reasons that may cause a wave of asbestos-related illnesses in Australia. The first wave was due to mining, whereas work and using asbestos products was responsible for the second wave.	The third wave is speculated to occur due to ambient exposure, using products, living with asbestos in building materials, and using asbestos-containing soils. The study advocates for the removal of asbestos from buildings as a sensible idea.
9	Castleman B [1]	2016	Asbestos mining and processing are happening in developing countries. Over 90% of the global asbestos is used in asbestos cement sheets and pipes, mostly for asbestos-cement roofing. Over 85% of consumption is in Asia.	There may be lobbying for doubting science, and mandatory standards for exposure could be discouraged. This calls for studies with high scientific integrity and implementation of the WHO mandate to stop the use of all types of asbestos.
10	Boulanger G, Andujar P, Pairon JC, et al. [15]	2014	The study reviewed various literature and highlighted the fact that the length of asbestos fibers above five microns alone linked to its adverse health effects may not be appropriate, and asbestos fibers with length below five microns, called SAF, are not only a good indicator of degradation of asbestos-containing materials but may also be a possible health indicator, especially when the indoor air quality is concerned.	The method of measurement in the past may need replacement by a newer method, and that will change the current standard for the length of fibers considered harmful to human health.
11	Zarogoulidis P, Orfanidis M, Constadinidis TC, et al. [13]	2011	In this case report, a 26-year-old person was diagnosed with mesothelioma, and the only exposure was of six years at the school, where asbestos-containing materials were used.	Even though control was absent, and the sample is small, we cannot rule out the possibility of non-occupational, pre-demolition and post-construction exposure as causes of disease.
12	Goldberg M, Luce D [14]	2012	This comprehensive study looks at multiple studies with pre-demolition and post-construction exposure. There is no epidemiological data with direct question of possible risk, but it is not possible to rule out harm.	The paper suggested an alternative approach, which assumes that since the incidence of mesothelioma in the absence of asbestos exposure cannot happen, all or most cases with mesothelioma, which are not exposed to occupational asbestos, are bound to be linked to non-occupational exposure.
13	Krakowiak E, Górny RL, Cembrzyńska J, Sakol G, Boissier-Draghi M, Anczyk E [12]	2009; Poland	This study suggests that we can map locate hot spots in a city. Additionally, it discovers that wind velocity, even at low levels, will disperse asbestos fibers from sources such as a building with asbestos cement sheets.	The study, conducted in Poland, shows that the urban and rural levels of ambient asbestos fiber concentrations in many countries, including India, was not available in literature.
14	Morinaga K, Kishimoto T, Sakatani M, et al. [11]	2001	In this study, no quantitative assessment of asbestos exposure was done, nor was there a focus on non-occupational exposure.	This study was included as it involved the tobacco use angle and the study could be linked to non- occupational exposure.
15	Mlynarek S, Corn M, Blake C [10]	1996	This study simply affirms that use of operations and maintenance protocols by building maintenance personnel decreased the risk of asbestos exposure.	There is a risk—even if not high—of exposure to building maintenance personnel.
16	Wilson R, Langer AM, Nolan RP, Gee JB, Ross M [7]	1994	This commentary text states that the risk of asbestos-containing materials may not be of real concern.	
17	Whysner J, Covello VT, Kuschner M, Rifkind AB, Rozman KK, Trichopoulos D, Williams GM [8]	1994	The study shuns the need for intervention in buildings that may have asbestos-containing materials, unless there is ongoing repair, etc.	
18	Brody AR [6]	1993	The study reinforces the belief that asbestos in all forms studied has harmful health impacts on humans. The study warns of using asbestos materials in buildings and the reason for continued exposure but does not investigate it further.	
19	Ganor E, Fischbein A, Brenner S, Froom P [5]	1992; Israel	A dining hall in a kibbutz was studied. It was found that the asbestos levels were higher than guideline requirement and suggested further research in all buildings where asbestos-containing materials are used.	
